# Valproate Use Is Associated With Posterior Cortical Thinning and Ventricular Enlargement in Epilepsy Patients

**DOI:** 10.3389/fneur.2020.00622

**Published:** 2020-07-02

**Authors:** Manuela Tondelli, Anna Elisabetta Vaudano, Sanjay M. Sisodiya, Stefano Meletti

**Affiliations:** ^1^Neurology Unit, OCSAE Hospital, AOU Modena, Modena, Italy; ^2^Department of Clinical and Experimental Epilepsy, UCL Queen Square Institute of Neurology, London, United Kingdom; ^3^Chalfont Centre for Epilepsy, Chalfont, United Kingdom; ^4^Department of Biomedical, Metabolic and Neural Science, University of Modena and Reggio Emilia, Modena, Italy

**Keywords:** valproate, epilepsy, brain morphometry, cortical thickness, brain structure

## Abstract

Valproate is a drug widely used to treat epilepsy, bipolar disorder, and occasionally to prevent migraine headache. Despite its clinical efficacy, prenatal exposure to valproate is associated with neurodevelopmental impairments and its use in children and adults was associated with rare cases of reversible brain atrophy and ventricular enlargement. To determine whether valproate use is related with structural brain changes we examined through a cross-sectional study cortical and subcortical structures in a group of 152 people with epilepsy and a normal clinical brain MRI. Patients were grouped into those currently using valproate (*n* = 54), those taking drugs other than valproate (*n* = 47), and drug-naïve patients (*n* = 51) at the time of MRI, irrespectively of their epilepsy syndrome. Cortical thickness and subcortical volumes were analyzed using Freesurfer, version 5.0. Subjects exposed to valproate (either in mono- or polytherapy) showed reduced cortical thickness in the occipital lobe, more precisely in the cuneus bilaterally, in the left lingual gyrus, and in left and right pericalcarine gyri when compared to patients who used other antiepileptic drugs, to drug-naïve epilepsy patients, and to healthy controls. Considering the subgroup of patients using valproate monotherapy (*n* = 25), both comparisons with healthy controls and drug-naïve groups confirmed occipital lobe cortical thickness reduction. Moreover, patients using valproate showed increased left and right lateral ventricle volume compared to all other groups. Notably, subjects who were non-valproate users at the time of MRI, but who had valproate exposure in the past (*n* = 27) did not show these cortical or subcortical brain changes. Cortical changes in the posterior cortex, particularly in the visual cortex, and ventricular enlargement, are present in people with epilepsy using valproate, independently from clinical and demographical variables. These findings are relevant both for the efficacy and adverse events profile of valproate use in people with epilepsy.

## Introduction

Valproate (VPA) is a drug widely used to treat epilepsy, bipolar disorder, and occasionally to prevent migraine headache. VPA is a first-line antiepileptic drug (AED) in the treatment of Genetic Generalized Epilepsies (GGE) with established efficacy for absence, tonic-clonic and myoclonic seizures (1–3). VPA is also particularly effective in reducing the photoparoxysmal response in various epilepsy syndromes (4–6), but the mechanism whereby photosensitivity is abolished is still unknown. Possible effects of VPA on occipital cortical hyperexcitability have been demonstrated in migraine (7–9), though its mechanisms of action are still debated. Despite its clinical efficacy, its use in females must be carefully evaluated since recent evidence shows that prenatal exposure to valproate is associated with neurodevelopmental impairments, impaired language and intellectual abilities and also with structural brain cortical changes, suggesting an effect of this drug on the prenatal brain (10, 11). In children and adults, valproate use was associated with rare cases of reversible brain atrophy and ventricular enlargement with accompanying cognitive impairment or parkinsonism (12–14). In Alzheimer's Disease, valproate use was also related to accelerated brain volume loss, in particular with ventricular enlargement and hippocampal atrophy, and greater cognitive impairment (15, 16). Recently, Pardoe and colleagues evaluated brain structural measures in seven patients with epilepsy taking valproate compared to normal controls and to people with epilepsy not taking valproate (all males): they found that total brain volume, white matter volume, and parietal cortical thickness were reduced in the valproate group relative to controls and non-valproate users; in addition, when comparing past-valproate users with patients who had never taken valproate, no differences in brain structures emerged, suggesting that valproate-related alterations were transient and reversible (17). The largest published neuroimaging study of epilepsy identified patterns of shared gray matter reduction across epilepsy syndromes, informing our understanding of epilepsy as a network disorder (18). However, this study had not investigated the possible effects of antiepileptic drugs on brain structures.

In this study, we analyzed structural MRI brain scans from a large cohort of people with epilepsy in order to determine whether VPA use is associated with cortical and subcortical brain changes.

## Methods

### Participants and Study Population

We retrospectively reviewed the entire cohort of people with epilepsy who underwent an MRI study for various research projects between May 2007 and April 2017 at the Department of Neuroscience (total of 354 patients). We included only subjects with normal structural brain MRI on conventional diagnostic protocol at 3 Tesla and absence of intellectual disability (full-scale IQ > 80) and psychiatric comorbidities. We excluded participants with a progressive disease (e.g., Rasmussen's encephalitis, progressive myoclonus epilepsy), malformations of cortical development, hippocampal sclerosis, tumors or previous neurosurgery, as well as patients with developmental and epileptic encephalopathies. We therefore focused on a final pool of 152 people with epilepsy. Sixty-five patients had a diagnosis of Genetic Generalized Epilepsy (GGE) and 87 patients had a diagnosis of focal epilepsy, according to the definitions of the Commission on Classification and Terminology of the International League Against Epilepsy (19, 20). Patients were categorized with a focal epilepsy according to interictal or ictal EEG abnormalities and concordant clinical seizure semiology. Participants were included in the GGE group if they presented with tonic-clonic, absence, or myoclonic seizures with generalized spike-wave discharges on EEG. All patients included in the study had a video-EEG recording/monitoring confirming the clinical diagnosis.

Patients were grouped into those currently taking valproate (VPA+), those who were not valproate users (VPA-), and drug-naïve patients at the time of MRI, irrespectively of their epilepsy syndrome.

**Figure 1:**
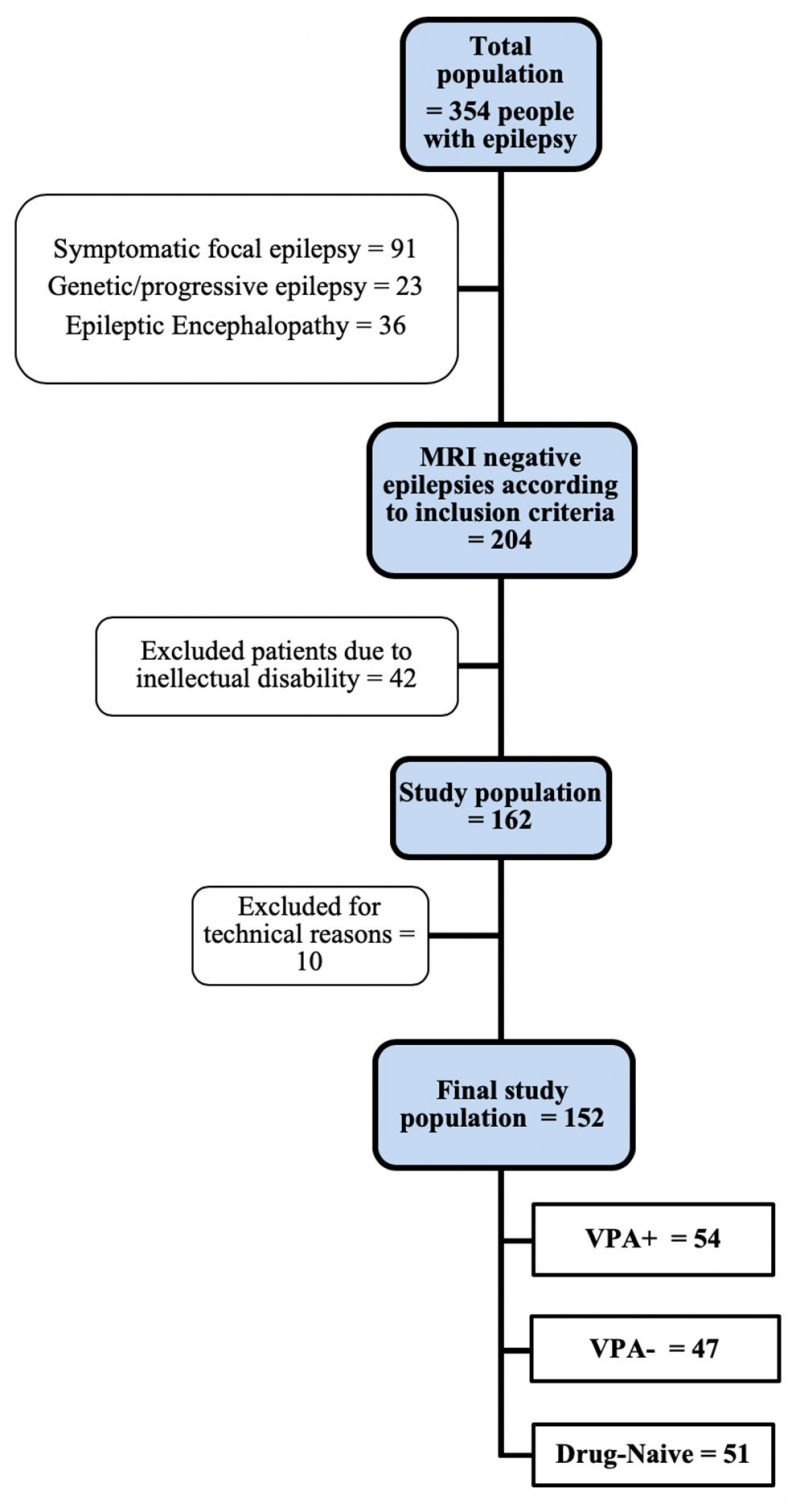
Study flow-chart. See text for inclusion/exclusion criteria. VPA+, patients using valproate at the time of MRI study. VPA-, patients using antiepileptic drugs other than valproate.

Figure 1 outlines the study flow-chart and the final study population.

For secondary analyses and in order to evaluate the effect of VPA use in mono- or polytherapy regimens, VPA+ patients were classified into subjects using VPA monotherapy and those taking VPA in polytherapy. No subject used drugs other than AEDs at the time of MRI.

To evaluate possible correlations between VPA exposure and brain morphometry measures, we recorded for each patient the VPA total daily dose, the plasma concentration of VPA, and the length of VPA exposure (months). Demographic data and clinical information such as duration of epilepsy, age of epilepsy onset, past antiepileptic drug prescriptions, type and number of AEDs at MRI, and treatment response were also collected for each patient.

For group comparisons, 40 volunteers were recruited as healthy controls (HC). HC had no history of neurological diseases or past valproate use, or family history of epilepsy, and had normal structural neuroimaging. Moreover, all controls had a normal EEG since they were recruited for previous EEG-fMRI co-registration study protocols by our group.

### MRI Acquisition

All subjects were studied with the same MRI scanner, sequences, and protocol across the study period. Three-dimensional (3D) T1-weighted MRI images were acquired using a 3 Tesla Philips Intera MRI scanner (Best, The Netherlands). A SPGR pulse sequence [echo time (TE) = 4.6, repetition time (TR) = 9.9 ms] was used. One hundred seventy contiguous sagittal slices were acquired (voxel size = 1 × 1 × 1 mm) and field of view was 240 mm with a matrix size of 256 × 256 × 170. A T2-weighted axial scan was also acquired to allow visual determination of vascular burden or tissue abnormalities.

### Statistical Analysis of Patients' Variables

Demographic and clinical characteristics of the subjects were analyzed using SPSS software 26.0 (SPSS Statistics, IBM, Chicago, IL). Independent samples *t*-tests were used to compare continuous variables and chi-square was used to compare categorical variables between groups.

### MRI Cortical Thickness and Subcortical Volume Analyses

Scans were analyzed using a standardized image toolbox (Freesurfer, version 5.0) (21), quality assurance (outlier detection based on inter quartile of 1.5 standard deviations along with visual inspection of segmentations), and statistical methods. Visual inspections of subcortical and cortical segmentations were conducted following standardized ENIGMA protocols (http://enigma.usc.edu), used in prior genetic studies of brain structure (22, 23), and large-scale case-control studies of epilepsy (18) and neuropsychiatric illnesses (24, 25). Analysts (MT; AEV) were blind to participant diagnoses. Briefly, as previously detailed the pipeline involves removal of non-brain tissue, automated Talaraich transformation, segmentation of white matter, and gray matter, tessellation of gray/white matter boundary, automated correction of topology defects, surface deformation to form the gray/white matter boundary and gray/cerebrospinal fluid boundary, and parcellation of cerebral cortex (26). Cortical thickness estimates were then calculated as the distance between the gray/white matter border and the pial surface at each vertex. Cortical thickness values were averaged over frontal, temporal, parietal, occipital, and insular lobes. In addition, single value labels extracted based on an automatic algorithm (27, 28) were calculated for more precise analyses (34 regions for each hemisphere). Subcortical volumes were calculated with FreeSurfer's automated procedure for volumetric measures. Each voxel in the normalized brain volume was assigned to one label using a probabilistic atlas obtained from a manually labeled training set (29). The labels adopted for the analysis included the putamen, caudate nucleus, globus pallidus, nucleus accumbens, thalamus, amygdala, hippocampus, and the ventricular system.

Statistical analyses were performed using SPSS software 26.0 (IBM, Chicago, IL). To compare cortical measures between groups, we conducted a univariate ANCOVA with each neuroanatomical value as the dependent variable, group diagnosis as fixed factor, and age, gender, intracranial volume (ICV), and disease duration (if applicable) as covariates. False discovery rate (FDR) was used to correct for multiple comparisons and a threshold of *p* < 0.05, estimated using SPSS, according to Benjamini and Hochberg methods (30), was considered statically significant.

## Results

One hundred fifty-two patients were finally evaluated for cortical and subcortical measures. Mean age of the patients at MRI considering the whole epilepsy group was 24 ± 5.2 years; mean disease duration was 8.2 years (± 3.4; range 0.5–10); 77 were females.

Table 1 reports the different study groups according to VPA use. Patients using VPA at the time of MRI (VPA+) were 54 (mean VPA dose = 900 mg/day). The median exposure time was 40 months (range: 1–250 months). The mean plasma concentration of VPA was 57.8 ug/ml (median 63 ug/ml; range 30–98 ug/ml). The VPA+ group consisted of 25 patients on monotherapy and 29 patients using VPA in polytherapy regimens (median number of AEDs = 2). There were 47 VPA- patients taking different AEDs in mono or polytherapy regimens (median number of AEDs = 2). Fifty-one patients were drug-naïve at the time of the MRI study. The number of AEDs and treatment response were comparable between VPA+ and VPA- groups. Antiepileptic drugs used by non-VPA users (*n* = 47) and by VPA+ patients in polytherapy are reported in Supplementary Table 1 (numbers of patients with specific drugs were too small to allow sub-analysis for other single specific AEDs).

**Table 1:** Demographic and clinical variables of the different groups according to valproate (VPA) treatment.

	**VPA users (VPA+)**	**non-VPA users (VPA-)**	**Drug-naïve**	**Healthy controls**	**p**
N. of subjects	54	47	51	40	
Female gender %	39%	53%	49%	52%	n.s.
Age (years), means ± sd	23.2 ± 4.7	30.2 ± 8.2	21.5 ± 2.7	25.5 ± 5.7	<0.01^*^
Disease duration (years), mean	8.6	12.6	4.9	–	<0.01^**^
Patients with GGE (%)	32 (59%)	9 (19%)	24 (47%)	–	<0.01^*^
Patients with Focal Epilepsy (%)	22 (41%)	38 (81%)	27 (53%)	–	<0.01^*^
Patients with TLE	4	17	7	–	
Patients with F/I/C lobe epilepsy	18	18	19	–	
Patients with P/O lobe epilepsy	0	3	1	–	
Patients on mono-therapy (%)	25 (46%)	15 (32%)	–	–	n.s.
Median number of AEDs (mode)	2 (1)	2 (1)	–	–	n.s.
Number of patients with drug-resistant epilepsy (%)	26 (48%)	28 (59%)	–	–	n.s.
Valproate mean daily dose (median), mg	900 (1,000)	–	–	–	

*VPA+, valproate users; VPA-, non-VPA users; TLE, temporal lobe epilepsy; F, frontal; I, insular; C, central; P, parietal; O, occipital*.

* *Non-VPA users were older and showed more frequently a “focal epilepsy phenotype” respect with other patients' groups*.

** *Drug-naïve patients had a shorter disease duration respect with other epilepsy groups*.

### Cortical Thickness Analyses According to Valproate Exposure

For all analyses, disease duration (apart from age, gender and ICV) was included as covariate of no interest to rule out a possible effect of this variable on brain structural results.

Direct comparison between VPA- (*n* =47) and HC groups, and between VPA- and “drug-naïve” patients (*n* = 51) did not reach any significant differences, either at the lobar level, or for any cortical parcellation.

**Figure 2:**
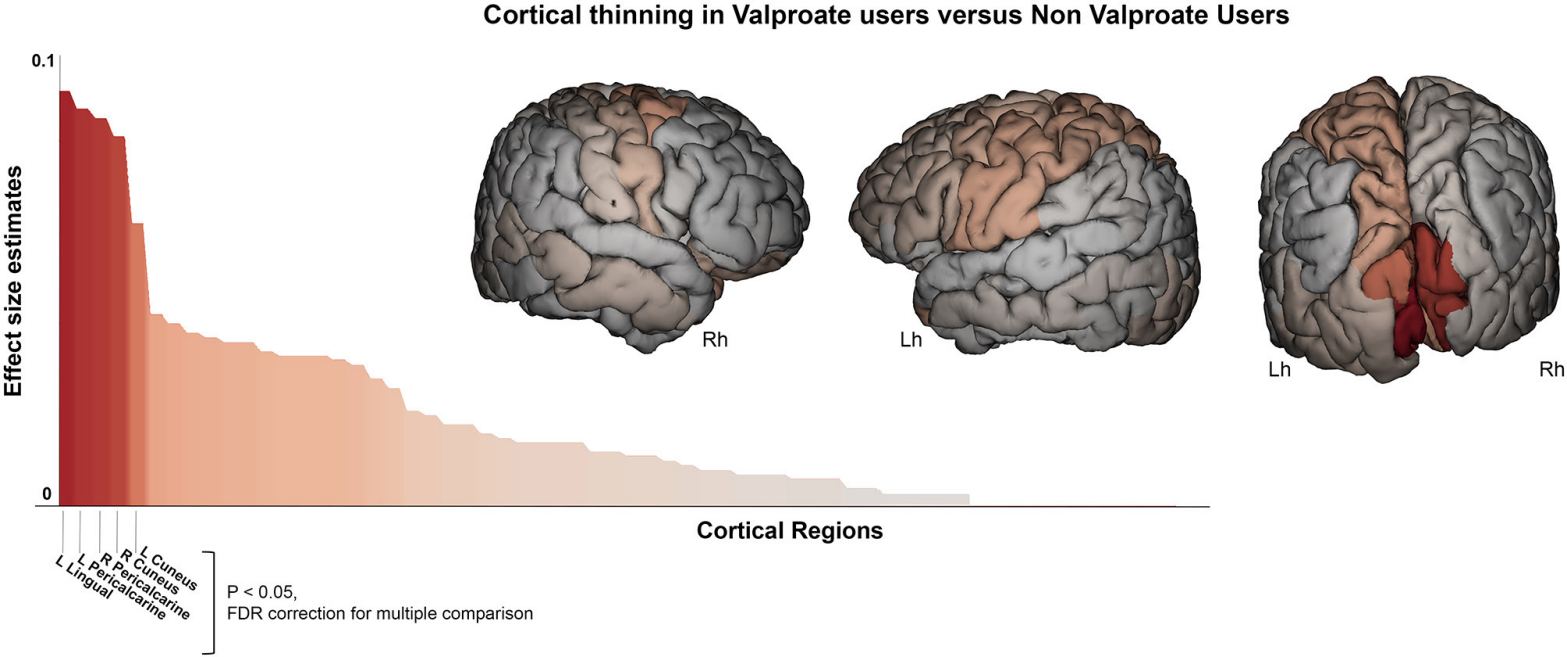
Cortical thickness difference between valproate users and non-valproate users. Surface brain template showing regions of cortical thinning in valproate users compared to non-valproate users. Brain images are generated using EnigmaViewer (https://www.nitrc.org/projects/enigmaviewer_20/); strength (color) of heat map is determined by the size of the regional effect size estimate. Effect size estimates (partial eta squared, y-axis) for cortical thickness differences across all brain regions is showed on the bar plots. Brain regions with significant differences between groups (*p* < 0.05 FDR; ANCOVA analysis adjusted for age, sex, disease duration, and intracranial volume) are reported on the x-axis. Lh, left hemisphere; Rh, right hemisphere.

Table 2 show the main results and the between-groups significant comparisons. The VPA+ group (*n* = 54) showed reduced cortical thickness compared to the VPA- group within the occipital lobe (*F* = 6, *p* = 0.016). More precisely, cortical thickness reduction was found in left (*F* = 5.6, *p* = 0.020) and right (*F* = 5.8, *p* = 0.019) cuneus, in left (*F* = 6.7, *p* = 0.014) and right (*F* = 5.5, *p* = 0.020) lingual gyrus, and in right (*F* = 5.5, *p* = 0.020) pericalcarine gyrus (Figure 2). We repeated the analysis adding also the “epilepsy type” (focal epilepsy/GGE) as covariate confirming these results (Supplementary Table 2).

**Table 2:** Significant cortical thickness analyses results.

**Group comparisons**	**Hemisphere**	**Cortical thickness measure (mm)**		***F***	***p***
**VPA+** **vs. VPA-**		**VPA+**	**VPA-**		
**Occipital lobe**		1.8992	1.9485	6	0.016
cuneus	L R	1.8462 1.8369	1.8956 1.8975	5.6 5.8	0.020 0.019
pericalcarine gyrus	L R	2.0250 2.0289	2.0499 2.0769	6.7 5.5	0.014 0.020
lingual gyrus	L	1.5884	1.6217	5.5	0.020
**VPA+** **vs. Drug-naive**		**VPA+**	**Drug-naive**		
**Occipital lobe**		1.8992	2.0298	20	0.000
cuneus	L R	1.8462 1.8584	2.0135 2.0478	10.7 15.9	0.001 0.000
lingual gyrus	L	2.025	2.1824	15	0.000
pericalcarine gyrus	L R	1.5668 1.5884	1.7397 1.7675	20 16	0.000 0.000
**VPA+** **vs. HC**		**VPA+**	**HC**		
**Occipital lobe**		1.8992	1.9632	11.5	0.001
pericalcarine gyrus	R	1.5884	1.6723	11.8	0.001
**VPA monotherapy vs. HC**		**VPA mono**	**HC**		
**Occipital lobe**		1.9315	1.9632	7	0.001
pericalcarine gyrus	R	1.603	1.6723	8.1	0.001
**VPA monotherapy vs. Drug-naive**		**VPA mono**	**Drug-naive**		
**Occipital lobe**		1.9315	2.0298	7.1	0.001
pericalcarine gyrus	L	1.6372	1.7397	14.5	0.000

*For each group comparison, only statistically significant cortical thickness measure (mm) for each subgroup are included; F and p value are reported as results of statistical analysis*.

*VPA+, valproate users; VPA-, non-VPA users; HC, healthy controls; Mono, monotherapy*.

**Figure 3:**
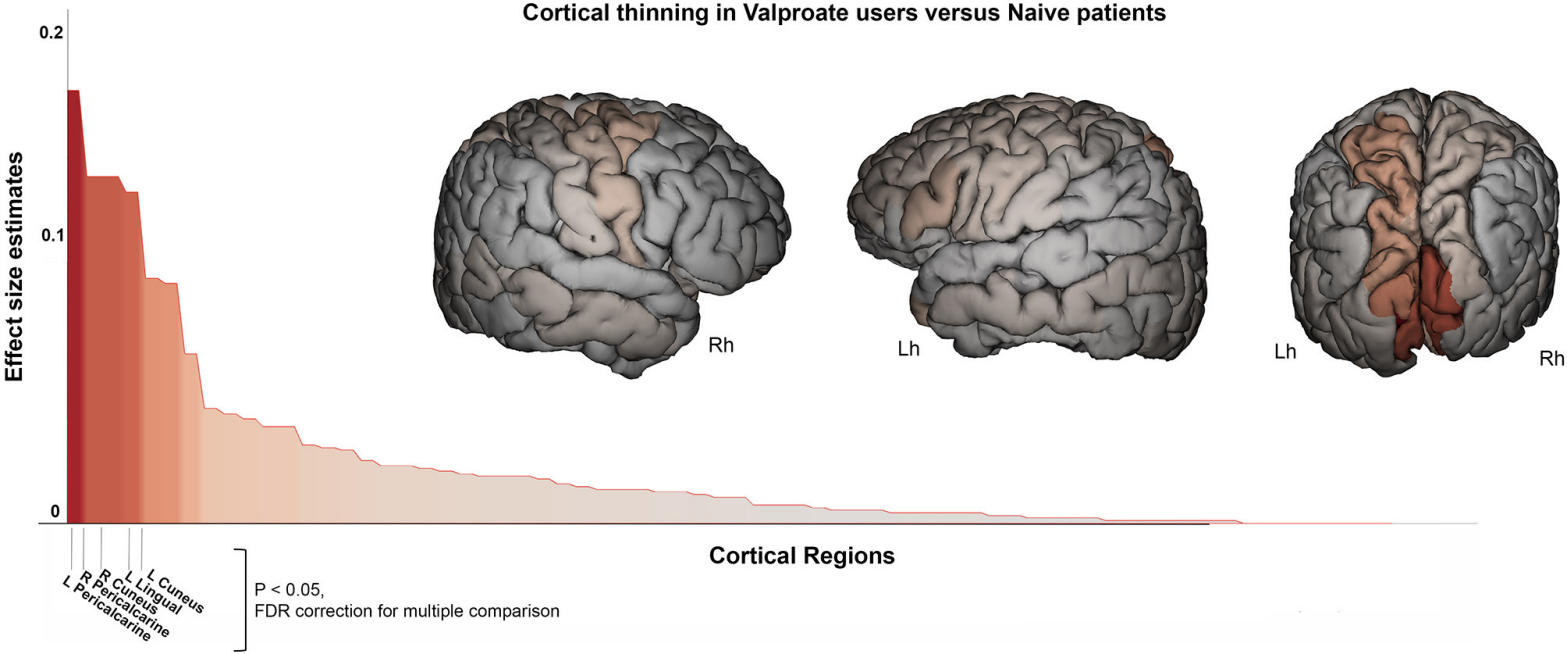
Cortical thickness difference between valproate users and drug-naïve patients. Surface brain template showing regions of cortical thinning in valproate users compared to drug-naïve patients. Brain images are generated using EnigmaViewer (https://www.nitrc.org/projects/enigmaviewer_20/); strength (color) of heat map is determined by the size of the regional effect size estimate. Effect size estimates (partial eta squared, y-axis) for cortical thickness differences across all brain regions is showed on the bar plots. Brain region with significant differences between groups (*p* < 0.05 FDR; ANCOVA analysis adjusted for age, sex, disease duration, and intracranial volume) are reported on the x-axis. Lh, left hemisphere; Rh, right hemisphere.

A similar pattern of cortical thickness differences emerged when comparing VPA+ vs. drug-naïve epilepsy patients (*n* = 51): this analysis revealed thickness reduction in occipital lobe (*F* = 20, *p* < 0.001). In particular in left (*F* = 10.7, *p* = 0.001) and right cuneus (*F* = 15.9, *p* < 0.001), in left lingual gyrus (*F* = 15, *p* < 0.001), in left (*F* = 20, *p* < 0.001) and right (*F* = 16, *p* < 0.001) pericalcarine cortex (Figure 3). Notably these two groups were matched for epilepsy type, without significant differences by syndrome (GGE or focal epilepsies). Occipital lobe cortical thickness reduction was also revealed in VPA+ compared to HC (*F* = 11.5, *p* = 0.001).

Finally, a subgroup analysis considering only patients using VPA monotherapy (*n* = 25) with respect to all other groups confirmed the occipital lobe cortical thickness reduction (in particular pericalcarine cortex) in VPA users (see Table 2).

Since dose-dependency has been reported in previous studies to increase the risks of congenital anomalies for VPA, and especially at doses above 800 mg/day (31), we also considered these possible dose-effects on cortical and subcortical brain measures. No correlations were found between cortical thickness measures and VPA total daily dose and VPA plasma concentration. Furthermore, no differences emerged when comparing VPA+ patients classified according to VPA total daily dose < or > 800 mg. Finally, no correlations were found between the length of VPA exposure (months) and cortical measures.

### Subcortical Structures Analysis According to Valproate Exposure

As for cortical measures, direct comparison between VPA- and healthy controls and between VPA- and drug-naïve patients did not show any significant differences for subcortical structures.

**Figure 4:**
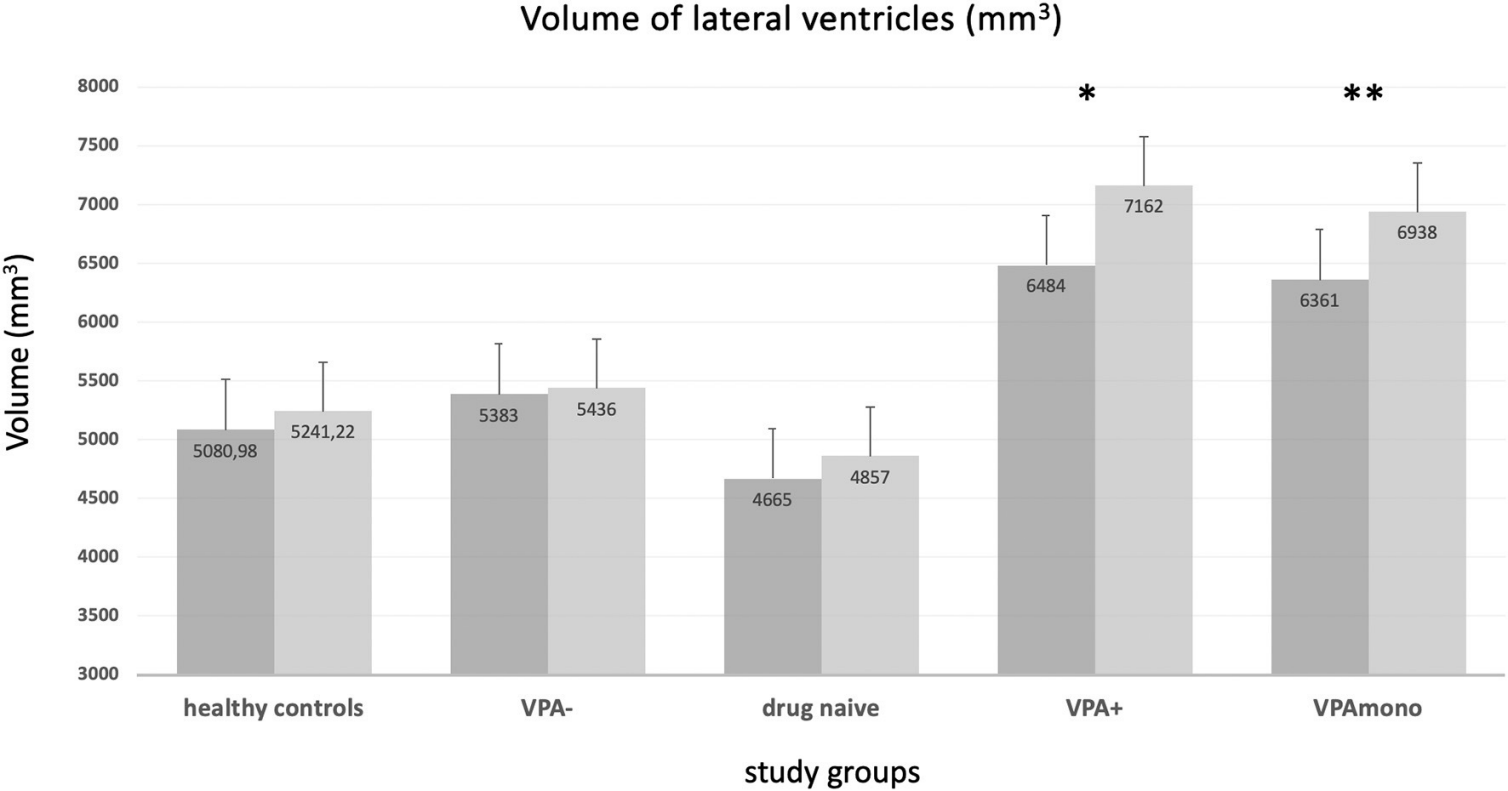
Volume of the lateral ventricles (means, mm^3^) in the different groups. VPA+ (^*^) and VPA mono (^**^) groups showed enlarged ventricular volumes (both right and left ventricle) with respect to every other group. No significant differences were present for healthy controls, VPA-, and drug-naïve groups (see text and Table 3). VPA+: epilepsy patients using valproate (in mono or polytherapy); VPA-, patients not using valproate; VPA mono, patients using valproate in monotherapy. Bars represent the standard error of the mean.

On the other hand, the VPA+ group showed increased left (*F* = 17, *p* < 0.001) and right lateral (*F* = 12.2, *p* = 0.001) ventricle volume compared to VPA- (Table 3, Figure 4). Left (*F* = 12, *p* = 0.001) and right lateral (*F* = 12, *p* = 0.001) ventricles were also larger in the VPA+ vs. drug-naïve patients. The direct comparison between VPA+ and HC showed increased left (*F* = 20.3, *p* < 0.001) and right (*F* = 20.9, *p* < 0.001) lateral ventricle volume and also a reduction in the volumes of the right pallidum (*F* = 11.4, *p* = 0.001) and left hippocampus (*F* = 12.2, *p* = 0.001). We repeated the analysis adding also epilepsy type as covariate and the results did not change (Supplementary Table 2). The same difference emerged when comparing the VPA monotherapy subgroup with HC and with drug-naïve patients.

**Table 3:** Significant results from subcortical structure analyses.

**Group comparisons**	**Hemisphere**	**Volume measure (mm**^****3****^**)**		**F**	**p**
**VPA+** **vs. VPA-**		**VPA+**	**VPA-**		
Lateral ventricle	L	7,162.71	5,436.5442	17	0.000
	R	6,484.076	5,382.4883	12.2	0.001
**VPA+** **vs. Drug-naive**		**VPA+**	**Drug-naive**		
Lateral ventricle	L	7,162.71	4,857.9319	12	0.001
	R	6,484.076	4,665.7277	12	0.001
**VPA+** **vs. HC**		**VPA+**	**HC**		
Lateral ventricle	L	7,162.71	5,241.2211	12	0.001
	R	6,484.076	5,080.9842	12	0.001
Pallidum	R	1,472.9045	1,551.3053	11.4	0.001
Hippocampus	L	4,188.306	4,585.5263	12.2	0.001
**VPA monotherapy vs. HC**		**VPA mono**	**HC**		
Lateral ventricle	L	6,938.2818	5,241.2211	7	0.001
	R	6,361.3636	5,080.9842	8	0.001
**VPA monotherapy vs. Drug-naive**		**VPA mono**	**Drug-naive**		
Lateral ventricle	L	6,938.2818	4,857.9319	6.4	0.001
	R	6,361.3636	4,665.7277	6	0.001

*For each group comparison, only statistically significant measure (mm^3^) for each subgroup are included; F and p value are reported as results of statistical analysis*.

*VPA+, valproate users; VPA-, non-VPA users; HC, healthy controls; Mono, monotherapy*.

As for cortical thickness analysis, no correlations were found between subcortical measures and VPA total daily dose or VPA plasma concentration. No differences emerged when considering VPA+ patients classified according to VPA total daily dose < or > 800 mg. No correlations were found between duration of VPA exposure (months) and subcortical measures.

### Past Exposure to Valproate

To evaluate possible long-term effects of VPA exposure on brain structure, we evaluated patients who were no longer VPA users at the time of MRI but who had been exposed to VPA in the past (*n* = 27; 11 females; mean age 24.2 ± 3.2; time since VPA withdrawal 6 months to 5 years). Cortical and subcortical measures of patients with past-exposure to VPA were compared to HC and to drug-naïve patients: there were no significant differences for any cortical brain parcellation or for any subcortical structure.

## Discussion

The main study finding showed occipital lobe cortical changes, particularly in the visual cortex, and ventricular enlargement in people with epilepsy using VPA compared to those not on VPA. These differences were independent of clinical and demographic variables. Moreover, a direct comparison between patients with past exposure to VPA and controls' groups did not reveal significant differences, suggesting that valproate-related changes might be transient and reversible. Overall, even if the retrospective cross-sectional design of this study cannot establish a causal role of VPA on the observed brain structural changes, the observed findings are presumably related to VPA use and are not driven by epilepsy *per se* or by generalized or focal epilepsy phenotype. Notably, these results are consistent with previous case reports of “pseudoatrophy” and ventricular enlargement associated with cognitive impairment or parkinsonism in children and adults with epilepsy (12–14) and with clinical trials evaluating the effect of valproate use in Alzheimer's disease patients, showing accelerated brain volume loss and ventricular enlargement in VPA users compared to non-users (15, 16).

Since its original discovery in France in early 60 s as an analog of valeric acid, sodium valproate has become one of the mainstays for the treatment of different epilepsy syndromes in adults and children (1). However, despite its long-standing usage, its multi-faceted mechanism of action is still matter of debate.

Sodium valproate increases gamma-aminobutyric acid (GABA) synthesis and release and hence potentiates GABAergic transmission in several brain regions. It also attenuates neuronal excitation mediated by activation of N-methyl-d-aspartate glutamate receptors. Moreover, a direct action on blocking of voltage-dependent sodium channels was also reported (1).

The occipital, and particularly the visual cortex changes related to VPA exposure are of particular interest considering the established efficacy of this drug in the treatment of GGE, and in particular to reduce the photoparoxysmal response in photosensitive epilepsies (6), as well as its efficacy in reducing cortical excitability in migraine (7). It is of interest that valproate is largely used in treating patients with electroclinical syndromes for whom the visual system has been demonstrated to be the hub of ictogenesis. One is eyelid myoclonia with absence syndrome (Jeavons syndrome) in which the photoparoxysmal response is a cardinal feature of the syndrome itself. Notably, an increased thickness of the visual cortex has been demonstrated in these patients (compared to healthy controls and patients without photosensitivity) as well as an increased functional connectivity between parieto-occipital networks and the motor system (32–34). Another such syndrome is idiopathic occipital lobe epilepsy, a focal idiopathic epilepsy syndrome of childhood, in which the extrastriate visual cortices are involved in spike generation and propagation (35). Moreover, valproate has been demonstrated to modulate (decrease) the excessive functional connectivity between parieto-frontal and motor networks in patients with juvenile myoclonic epilepsy, the commonest GGE syndrome in adolescent and young adults, indicating a possible normalizing effect of this drug (36). Recently, reduced functional connectivity in primary visual and parietal brain networks was noted after administration of VPA in functional neuroimaging studies in the baboon model of genetic generalized epilepsy, suggesting a presumed therapeutic benefit in terms of seizure generation inhibition (37).

Considering the clinical effects of VPA on migraine and photosensitivity and the present findings of VPA-related thinning of posterior cortical regions, we hypothesize that some components of the mechanism of action of VPA are related to cortical structural changes, whether related to its action on GABA or ion-channels neurotransmission or not. Interestingly, vigabatrin, an irreversible GABA-transaminase inhibitor used as first-line treatment in children with infantile spasms, has been demonstrated to induce reversible subcortical alterations (age- and dose-related) in young infants (38, 39). The most common sites for abnormal MRI signal intensity and/or restricted diffusion were the basal ganglia, followed by the dorsal brainstem, dentate nuclei, and thalami. No study to our knowledge has investigated this effect by means of advanced quantitative MRI methods.

Although growing evidence points to a probable role of VPA in causing structural and functional brain changes, mechanisms underlying these changes are still poorly understood. A possible explanation involves inhibition of neurite outgrowth (40), but also osmotic changes (41), and neurotoxicity (42) have been hypothesized. Another proposed mechanism is through mitochondrial dysfunction, as suggested in patients with mitochondrial DNA mutation and VPA toxicity (43). More recently, converging evidence suggests a possible epigenetic effect of VPA on human central nervous system, mediated by histone deacetylase (HDACs) dysregulation (44). Indeed, systemic administration of valproate, a recognized global HDACs inhibitor, induced myelination dysregulation in animals' brain (45, 46), suggesting that chronic exposure to valproate may profoundly affect this process (47). Moreover, VPA treatment produced marked alterations in the expression of multiple genes, many of which are involved in transcription regulation, cell survival, ion homeostasis, cytoskeletal modifications and signal transduction (48). As suggested by Rosenzweig et al. (47) “VPA via its biochemical, molecular and epigenetic mechanisms may simultaneously act to promote neuronal survival and proliferation, while also negatively affecting differentiation of local oligodendrocyte progenitor cells.” The advent of several new imaging techniques, including DTI or myelin-specific MRI techniques, will enable *in vivo* visualization of any such effects of VPA. Unfortunately, in our study we did not collect specific MRI sequences to analyse white matter or tractography, but we can speculate that our finding of bilaterally larger lateral ventricle volume in patients taking VPA in comparison to controls' groups might reflect a global white matter change related to VPA role as pan-HDACs inhibitor of (re)myelination efficiency and homeostasis. Importantly, epigenetic effects are related to the duration of exposure to VPA. Therefore, the putative effects of valproate as an HDAC inhibitor could also explain the reversibility of the cortical and subcortical structural changes that we observed in the patients that were no longer VPA users.

### Study Limitations

This study has several limitations. The first one as already pointed is the retrospective and cross-sectional design of the study. Therefore, a direct causation of VPA on the observed brain structural changes cannot be established. A prospective within-subject longitudinal study should be performed to address this question. Second, we did not collect neurobehavioral measures. This prevents to correlate cortical changes revealed by MRI and neuropsychological and behavioral features, as suggested by previous case reports (10, 14). Other limitations of this study concern the sample size of the different groups: even though the sample was larger with respect to previous MRI studies investigating AEDs effects on brain structure, it was still limited. However, it should be noted that detailed AED information is typically difficult to obtain in larger and multicentre retrospective datasets. Indeed, the only other study that evaluated with structural imaging the VPA effect in people with epilepsy included only seven/nine subjects and all were male (17). Finally, the duration of the disease was significantly different between groups, being shorter in drug-naïve patients and longer in other groups. To mitigate the influence of disease duration, we treated this variable as a confound in all statistical analyses. Moreover, the VPA users had shorter disease duration with respect to non-VPA users, suggesting that it is highly improbable that the observed VPA-related findings are a consequence of a longer disease duration in patients exposed to VPA.

### Conclusion

We observed that occipital lobe cortical changes, particularly in the visual cortex, are present in people with epilepsy using VPA. These differences were independent from clinical and demographic variables, and specific for VPA use. The fact that these changes were not observed in patients with past exposure to valproate may suggest reversibility. We believe these findings are relevant both in relation to efficacy and to the adverse events profile of VPA use in people with epilepsy. Finally, the findings of the present study should also be taken into account as a potential confounding factor in any MRI morphometric study in which subjects taking VPA are included.

## Data Availability Statement

The raw data supporting the conclusions of this article will be made available by the authors, without undue reservation.

## Ethics Statement

The human ethics committee of the University of Modena and Reggio Emilia approved this study and written informed consent was obtained from all subjects.

## Author Contributions

SM and MT: study design, drafting, and revising the manuscript. AV: data collection, data interpretation, and revising the manuscript. SS: data interpretation, drafting, and revising the manuscript. All authors contributed to discussion of the results and read and approved the final version of the manuscript.

## Conflict of Interest

SS has received honoraria or institutional grant support from UCB and Eisai. SM received research grant support from the Ministry of Health (MOH); has received personal compensation as scientific advisory board member for UCB and Eisai. The remaining authors declare that the research was conducted in the absence of any commercial or financial relationships that could be construed as a potential conflict of interest.
