# Supplementary material for: Valproate Use Is Associated With Posterior Cortical Thinning and Ventricular Enlargement in Epilepsy Patients
Source: Front Neurol. 2020 Jul 2;11:622. doi: 10.3389/fneur.2020.00622 (PMC7351506; doi:10.3389/fneur.2020.00622)
Supplement: Supplementary file 1 [file Data_Sheet_1.docx]

**Supporting Documents**

**Supplementary Table 1.**

Antiepileptic drugs used by non-valproate (non-VPA) users and by patients using valproate in polytherapy.

| **Drug** | **VPA polytherapy (n=29)** | **Non-VPA users (n=47)** |
| --- | --- | --- |
| Levetiracetam | 9 | 8 |
| Phenobarbital | 6 | 10 |
| Carbamazepine | 3 | 11 |
| Lamotrigine | 6 | 9 |
| Phenytoin | 1 | 6 |
| Clobazam | 3 | 6 |
| Lacosamide | 1 | 3 |
| Zonizamide | 1 | 1 |
| Etosuccimide | 3 | 0 |
| Topiramate | 1 | 1 |

**Supplementary Table 2. Significant cortical thickness and subcortical analyses results** **with ‘epilepsy type’ added as covariate**. For each group comparison, only statistically significant cortical thickness measure (mm) for each subgroup are included; F and p value are reported as results of statistical analysis.

| **Group comparisons** | **Hemisphere** | **Cortical thickness measure (mm)** | | **F** | **p** |
| --- | --- | --- | --- | --- | --- |
| **VPA+ vs VPA-** |  | **VPA+** | **VPA-** |  |  |
| **Occipital lobe** |  | 1.8992 | 1.9485 | 8.05 | 0.006 |
| cuneus | L | 1.8462 | 1.8956 | 7.67 | 0.007 |
| **Subcortical** |  |  |  |  |  |
| Lateral ventricle | L | 7162.71 | 5436.5442 | 4.87 | 0.031 |
|  |  |  |  |  |  |
| **VPA+ vs Drug-naive** |  | **VPA+** | **Drug-naive** |  |  |
| **Occipital lobe** |  | 1.8992 | 2.0298 | 11.6 | 0.001 |
| cuneus | R | 1.8584 | 2.0478 | 11.4 | 0.001 |
| lingual gyrus | L | 2.0251 | 2.1824 | 9.16 | 0.003 |
| pericalcarine gyrus | L  R | 1.5668  1.5884 | 1.7397  1.7675 | 14.2  10.5 | 0.000  0.002 |
| **Subcortical** |  |  |  |  |  |
| Lateral ventricle | L | 7162.71 | 4857.9319 | 11 | 0.001 |
|  | R | 6484.076 | 4665.7277 | 13 | 0.000 |

VPA+: valproate users. VPA-: non-VPA users.
